# Long-term safety and effectiveness of growth hormone therapy in Korean children with growth disorders: 5-year results of LG Growth Study

**DOI:** 10.1371/journal.pone.0216927

**Published:** 2019-05-16

**Authors:** Young-Jun Rhie, Jae-Ho Yoo, Jin-Ho Choi, Hyun-Wook Chae, Jae Hyun Kim, Sochung Chung, Il Tae Hwang, Choong Ho Shin, Eun Young Kim, Ho-Seong Kim

**Affiliations:** 1 Department of Pediatrics, Korea University College of Medicine, Ansan, Korea; 2 Department of Pediatrics, College of Medicine, Dong-A University, Busan, Korea; 3 Department of Pediatrics, Asan Medical Center, University of Ulsan College of Medicine, Seoul, Korea; 4 Department of Pediatrics, Yonsei University College of Medicine, Seoul, Korea; 5 Department of Pediatrics, Seoul National University Bundang Hospital, Seongnam, Korea; 6 Department of Pediatrics, Konkuk University School of Medicine, Seoul, Korea; 7 Department of Pediatrics, College of Medicine, Hallym University, Seoul, Korea; 8 Department of Pediatrics, Seoul National University College of Medicine, Seoul, Korea; 9 LG Chem, Ltd., Seoul, Korea; University of South Alabama Mitchell Cancer Institute, UNITED STATES

## Abstract

**Purpose:**

The aim of this registry study was to analyze the long-term safety and effectiveness of recombinant human growth hormone (rhGH) in South Korean pediatric patients (≥2 years of age) with growth hormone deficiency GHD) of idiopathic or organic etiology, idiopathic short stature, Turner syndrome, small for gestational age and chronic renal failure.

**Methods:**

The study patients were followed-up till two years after the epiphyseal closure, with visits scheduled every six months. The outcome measures included the incidence of adverse events (AEs, in particular, neoplasia, glucose intolerance and hypothyroidism), as well as height standard deviation score (Ht SDS) and annual height velocity. The results of the interim analysis of a 5-year accumulated data for 2,024 patients (7,342 patient-years, PY) are presented.

**Results:**

A total of 14 neoplasms were diagnosed (191/100,000 PY); 7 out of 9 malignancies were recurrent craniopharyngioma found in patients with organic GHD. Seven cases of glucose intolerance (95/100,000 PY) and 22 cases of hypothyroidism (300/100,000 PY) were detected; about half of the cases (4 and 10 cases each) were considered to be related with rhGH treatment. Most of the growth-retarded patients showed continuous improvement in Ht SDS, with the most prominent effect observed within a year of treatment initiation. The beneficial effect of rhGH on Ht SDS gain was maintained for 2–4 years.

**Conclusions:**

The incidence of AEs of interest in rhGH-treated patients was low, and most of the neoplasms were benign and/or non-related to rhGH. Most patients benefited from the therapy in terms of height increment.

## Introduction

Nowadays, indications for treatment with recombinant human growth hormone (rhGH) include not only growth hormone deficiency (GHD) but also a number of other conditions, such as Turner syndrome (TS), Prader-Willi syndrome (PWS), small for gestational age (SGA), chronic renal failure (CRF) and idiopathic short stature (ISS) [1–5]. Although rhGH therapy is generally considered to be safe, still there are safety issues that need to be addressed, i.e., the risk of neoplasia and cardiovascular mortality [6–8]. Another important question is the long-term effectiveness of rhGH in various indications. This subject is rarely explored in clinical trials conducted for a purpose of product registration, which usually have relatively short follow-up periods.

To overcome these knowledge gaps, several large-scale registry studies of rhGH-treated patients have been initiated [9–11]. In addition, pharmaceutical companies carried out research on the safety and effectiveness of their products in various populations [12–15]. However, the efficacy and safety of rhGH have not been investigated in a large number of Asian patients. Thus, we designed a registry study (LG Growth Study, LGS) to analyze the long-term safety and effectiveness of rhGH (Eutropin inj., Eutropin AQ inj., Eutropin Pen inj. and Eutropin Plus inj., LG Chem, Ltd., Korea) in patients with GHD, ISS, TS, SGA and CRF. Detailed descriptions of the individual rhGH products and study background are provided in the previous publication introducing the study protocol and cohort characteristics [16]. In this paper, we present the results of the interim analysis of a 5-year accumulated data from the LGS.

## Methods

### Patients and study design

LGS is a multi-center (total 73 sites), non-interventional registry study to evaluate the long-term safety and effectiveness of four rhGH products: a weekly rhGH (Eutropin Plus inj.) and three daily rhGHs (Eutropin inj., Eutropin AQ inj. and Eutropin Pen inj.) in patients ≥2 years of age with GHD, ISS, TS, SGA, or CRF. Patients treated with one of the aforementioned products were prospectively followed-up till two years after the epiphyseal closure. If patients who had been already on treatment were registered, the preregistration data were collected retrospectively. A set of minimal eligibility criteria was applied for each indication to register eligible patients, and approved product leaflets were referenced for choosing the initial dosage of rhGH [16]. When GH stimulation test was indicated, the investigator chose the appropriate test(s) among commonly used methods in the clinic, such as insulin tolerance test, L-dopa test, clonidine test, or glucagon test. No interventional element was applied under the study protocol. All treatment-related decisions were left at an investigator’s discretion to collect the data from real-life clinical practice.

Measurements of height, bone age, insulin-like growth factor I (IGF-I), IGF binding protein 3 (IGFBP-3), thyroid stimulating hormone (TSH), total thyroxine (T4), free T4 (fT4), hemoglobin A1c (HbA1c) and serum glucose levels were recorded at 6-month intervals. No central laboratory was used; all laboratory analyses were carried out according to local standard procedures of each site. IGF-1 SDS and IGFBP-3 SDS were calculated based on normative data for Korean population [17]. Adverse events (AEs), including AEs of interest (such as neoplasia, glucose intolerance and hypothyroidism), were collected through interviews and/or chart reviews. To maintain the real-life setting, all decisions regarding choice of rhGH product, its dosage and titration were solely at the investigator’s discretion, whereas the methodology of data collection was standardized to guarantee the integrity of the results. The study was conducted in accordance with the principles expressed in the Declaration of Helsinki and applicable regulations. The study protocol and consent form were reviewed by the institutional review board of each medical center, when required, and the list of institutional review boards that approved the study is provided in S1 Table. Written informed consent was obtained from the patients and/or their parents/legal representatives. The study was registered at ClinicalTrials.gov (identifier: NCT01604395).

### Statistical analysis

All patients treated at least once with one of the target products were included in the safety analysis. The incidence of AEs was expressed per 100,000 patient-years (PY). To calculate total PY, each patient’s time was summed up from the first date of rhGH treatment to the earliest date of the last follow-up, withdrawal, death or data cut-off. AEs were coded using MedDRA software ver. 17.0 (International Federation of Pharmaceutical Manufacturers Associations, http://www.meddra.org). Adverse drug reactions (ADRs) were classified as AEs with definite, probable, possible or unassessable causative relationship with rhGH.

Patients who did not satisfy the indication criteria, received rhGH for off-label treatment or had incomplete information for baseline or post-treatment height measurements were excluded from the effectiveness analysis. Height standard deviation score (Ht SDS) and annual height velocity (HV) were calculated from height records at protocol defined timepoints (with ± 1-month window period). To calculate Ht SDS, the following equation was used referencing the Box-Cox transformation (L), median (M), and coefficient of variation (S) values from growth standard for Korean children and adolescents [18]: height SDS = [Power (measured height/M, L)– 1] / L×S.

The data cut-off point was 22 March 2017. The study data and the results of the interim analysis were evaluated by the dedicated Observational Study Monitoring Board. The results for CRF cohort (n = 9) were included in the whole dataset, but are not presented separately in the effectiveness aspect due to a small sample size. Missing data were not substituted. Results with the *P*-values <0.05 were considered statistically significant. All statistical analyses were performed using SAS software version 9.4 (SAS Institute, Cary, NC, USA).

## Results

### Patients characteristics

**Fig 1:**
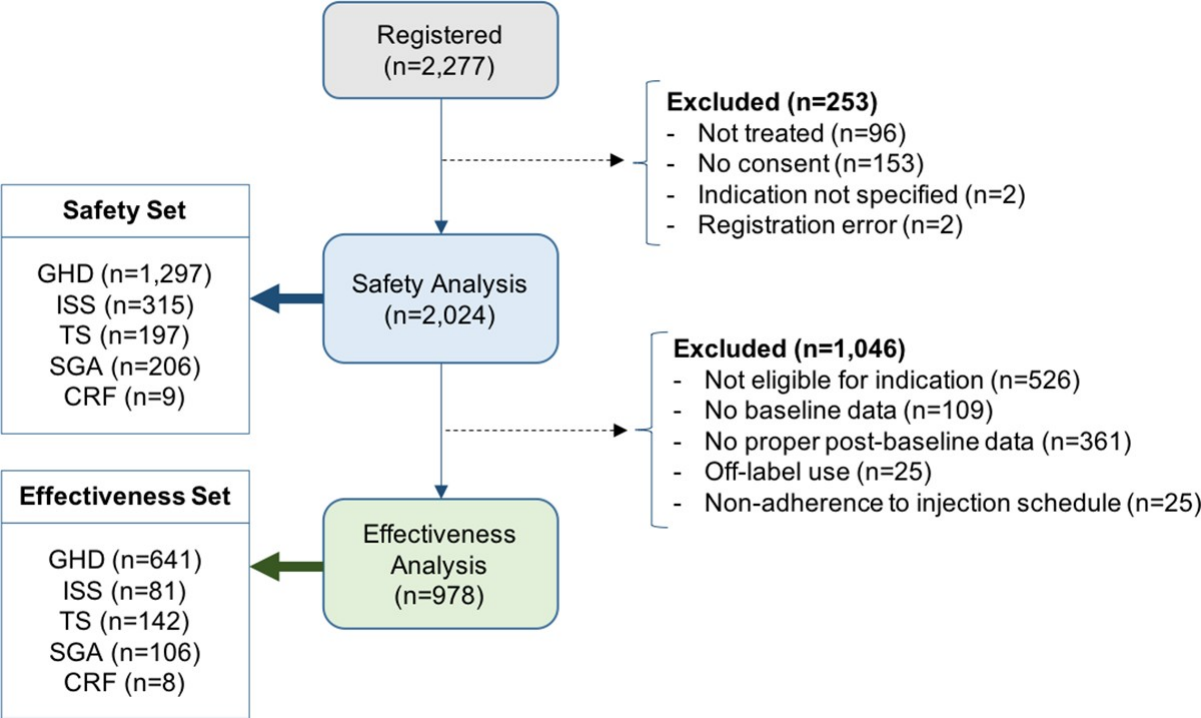
Disposition of patients.

A total of 2,277 patients were registered between November 2011 and March 2017. The safety data were available for 2,024 patients, which corresponds to 7,342 PY. Other 253 patients were excluded from the safety analysis because of missing treatment record or indication information, lack of consent or registration error (Fig 1). Median age at the time of registration was 8.49 years (range 2.01 to 19.23 years); 82.9% (male: 89.9%, female: 78.5%) patients were prepubertal (Tanner stage I). Other baseline characteristics are presented in Table 1. The most common indication for rhGH treatment was GHD (64.1%); the vast majority of GHD patients had idiopathic GHD (IGHD) (n = 1,189, 91.7%). Median Ht SDS in the study population was -2.26. Median Ht SDS at the baseline was the lowest in TS cohort (-2.58), and the most severe bone age (BA) delay (BA–chronological age) was observed in GHD cohort (median -1.71 years).

**Table 1:** Baseline demographic characteristics of patients.

Variable	Total (n = 2,024)	GHD (n = 1,297)	TS (n = 197)	SGA (n = 206)	ISS (n = 315)	CRF (n = 9)
Sex:						
male	1048 (51.8%)	771 (59.4%)	0 (0.0%)	104 (50.5%)	167 (53.0%)	6 (66.7%)
female	976 (48.2%)	526 (40.6%)	197 (100.0%)	102 (49.5%)	148 (47.0%)	3 (33.3%)
Age (years)	8.49 (2.01, 19.23)	8.42 (2.10, 19.23)	9.24 (2.01, 18.58)	6.66 (3.04, 14.18)	9.05 (2.38, 16.10)	8.58 (2.48, 15.88)
Puberty^a^						
pre-pubertal	793 (82.9%)	502 (87.3%)	107 (83.0%)	77 (83.7%)	105 (66.0%)	2 (100.0%)
pubertal	164 (17.1%)	73 (12.7%)	22 (17.1%)	15 (16.3%)	54 (34.0%)	0 (0.0%)
BA (years)	6.8 (0.6, 17)	6.0 (0.6, 17)	8.5 (1.0, 14)	5.5 (1.5, 13.5)	8.4 (2.0, 15.6)	8.8 (1.5, 13.5)
BA-CA (years)	-1.46 (-8.83, 4.49)	-1.71 (-8.83, 2.71)	-0.87 (-5.75, 2.53)	-1.05 (-4.67, 2.51)	-0.98 (-5.30, 4.49)	-1.69 (-4.87, 0.30)
Height SDS	-2.26 (-6.97, 6.23)	-2.25 (-6.97, 6.23)	-2.58 (-6.34, -0.66)	-2.23 (-5.14, -0.18)	-2.17 (-5.31, 1.54)	-2.43 (-3.54, 0.00)
BMI SDS	-0.27 (-16.88, 3.47)	-0.23 (-16.88, 2.88)	0.51 (-1.92, 3.47)	-0.81 (-5.60, 2.09)	-0.58 (-4.60, 2.69)	-0.89 (-2.89, 1.61)

a Some patients’ puberty data are missing and only available data are accounted.

Data show numbers (%) or medians (min, max).

*BA* bone age, *BMI* body mass index, *CA* chronological age, *CRF* chronic renal failure, *GHD* growth hormone deficiency, *ISS* idiopathic short stature, *SDS* standard deviation score, *SGA* small for gestational age, *TS* Turner syndrome.

Out of 2,024 patients included in the safety set, 1,046 were excluded from the effectiveness analysis because of non-eligibility for indications, missing baseline or follow-up data, off-label use and non-adherence (Fig 1). Demographic characteristics of the remaining 978 patients included in the effectiveness set resembled those of the safety set (S2 Table). The upper quartile for Ht SDS was -2.07, which implies that >75% of patients were severely growth-retarded.

### Use of rhGH products

Approximately 80% of the study patients were initially treated with a daily rhGH (n = 1,626, Eutropin inj. or Eutropin AQ inj.) whereas others were treated with a weekly rhGH (n = 398, Eutropin Plus inj.). Median (IQR) on-treatment follow-up period was 3.03 years (1.67, 4.65): 2.87 years (1.56, 4.51) for Eutropin inj., 2.29 years (0.9, 4.02) for Eutropin Plus inj. and 0.89 years (0.51, 1.52) for Eutropin AQ inj.. No significant changes were noted in the yearly descriptive statistics for rhGH doses in each indication. The 5-year overall median (IQR) dose of Eutropin inj. for GHD, TS, SGA, ISS and CRF cohorts was 0.24 mg/kg/week (0.21, 0.27), 0.31 mg/kg/week (0.27, 0.33), 0.27 mg/kg/week (0.22, 0.31), 0.26 mg/kg/week (0.21, 0.29) and 0.29 mg/kg/week (0.25, 0.31), respectively. Median (IQR) dose of Eutropin Plus inj. for GHD cohort was 0.60 mg/kg/week (0.53, 0.73). Statistical characteristics of rhGH dose for the effectiveness set were similar. Median (IQR) on-treatment follow-up period for the effectiveness set was 3.60 years (2.08, 5.10): 3.71 years (2.35, 5.14), 2.32 years (1.27, 3.82), 4.84 years (3.53, 7.08) and 1.99 years (1.44, 2.59) years for GHD, ISS, TS and SGA cohorts, respectively.

### Adverse events during on-treatment follow-up period

During on-treatment follow-up period (6,898 PY), 954 AEs (13,831 per 100,000 PY) were reported in 458 patients (22.6%). Proportions of patients experiencing AEs, ADRs, SAEs, and serious ADRs are provided in the Table 2. The following results are based on the number of events (cases). Most AEs (97.7%) were mild (772 cases) or moderate (160 cases) in severity, and 80.4% resolved. The most frequent AEs were upper respiratory tract infections (1,305 per 100,000 PY), typically unrelated to rhGH treatment. Out of 954 AEs, 119 (12.5%) were ADRs (1,725 per 100,000 PY). The most common type of ADRs were reactions in the injection site (25 cases, 362 per 100,000 PY), and among them, injection site pain (13 cases, 188 per 100,000 PY). Other common ADRs were headache (15 cases, 217 per 100,000 PY), hypothyroidism (10 cases, 145 per 100,000 PY) and arthralgia (7 cases, 101 per 100,000 PY). The highest incidence of AEs (per 100,000 PY) was documented in ISS cohort (18,745), followed by TS (17,885), SGA (15,179) and GHD (11,796) cohorts, and the highest incidence of ADRs (per 100,000 PY) in ISS cohort (2,734), followed by SGA (2,039), GHD (1,605) and TS (1,346) cohorts. Approximately 10% of AEs (94 cases) were serious AEs (SAEs), and 8 of them (116 per 100,000 PY) were regarded as rhGH-related, autoimmune thyroiditis, hypothyroidism, diabetes mellitus, hematuria, intervertebral disc protrusion, supraventricular tachycardia (1 case each) and craniopharyngioma (2 cases). No mortality was reported during the on-treatment follow-up period.

**Table 2:** Incidence of adverse events during on-treatment follow-up and whole study period.

Variable	Total (n = 2,024)^a^	IGHD (n = 1,189)	OGHD (n = 107)	TS (n = 197)	SGA (n = 206)	ISS (n = 315)	CRF (n = 9)
On-treatment follow-up period:							
AEs	458 (22.6%)	231 (19.4%)	45 (42.1%)	72 (36.6%)	35 (17.0%)	70 (22.2%)	5 (55.6%)
ADRs	93 (4.6%)	43 (3.6%)	13 (12.2%)	11 (5.6%)	8 (3.9%)	17 (5.4%)	1 (11.1%)
SAEs	66 (3.3%)	31 (2.6%)	12 (11.2%)	13 (6.6%)	4 (1.9%)	6 (1.9%)	-
Serious ADRs	7 (0.4%)	4 (0.3%)	2 (1.9%)	1 (0.5%)	-	-	-
Whole study period:							
AEs	462 (22.8%)	232 (19.5%)	46 (43.0%)	73 (37.1%)	35 (17.0%)	70 (22.2%)	6 (66.7%)
ADRs	94 (4.6%)	43 (3.6%)	14 (13.1%)	11 (5.6%)	8 (3.9%)	17 (5.4%)	1 (11.1%)
SAEs	68 (3.4%)	31 (2.6%)	13 (12.2%)	13 (6.6%)	4 (1.9%)	6 (1.9%)	1 (11.1%)
Serious ADRs^*^	8 (0.4%)	4 (0.3%)	3 (2.8%)	1 (0.5%)	-	-	

a Addition of each subgroup number does not sum up to 2,024 as one patient with GHD could not be classified as either IGHD or OGHD. The patient with non-specified GHD etiology did not experience any adverse event. Data show numbers (%) of patients with events.

^*^Total of nine serious ADRs were reported in eight patients during whole study period: autoimmune thyroiditis, hypothyroidism, diabetes mellitus, hematuria, intervertebral disc protrusion and supraventricular tachycardia (n = 1 each) and craniopharyngioma recurrence (n = 3). Except one case of craniopharyngioma recurrence, all occurred during on-treatment follow-up period.

*ADR* adverse drug reaction, *AE* adverse event, *CRF* chronic renal failure, *GHD* growth hormone deficiency, *IGHD* idiopathic growth hormone deficiency, *ISS* idiopathic short stature, *OGHD* organic growth hormone deficiency, *SAE* serious AE, *SGA* small for gestational age, *TS* Turner syndrome.

### Adverse events during off-treatment follow-up period

Off-treatment follow-up has been completed or ongoing in 335 patients. Median off-treatment follow-up duration was 1.0 year (range 0 to 9.55 years). During this period, 26 additional AEs (25 non-related to rhGH and 1 ADR) occurred including 5 SAEs. One SAE (craniopharyngioma recurrence) was evaluated as rhGH-related. A 12-year-old male patient from IGHD cohort, with unremarkable medical history, was diagnosed with medulloblastoma during the rhGH treatment (approximately one year after the start of the rhGH treatment). At the baseline, the patient’s IGF-I (SDS) and IGFBP-3 (SDS) were 161 ng/mL (-0.84) and 5,280 ng/mL (3.83), respectively, and the initial dosage was 0.28 mg/kg/week with Eutropin inj. About 6 months later, IGF-I (SDS) and IGFBP-3 (SDS) were 257 ng/mL (-0.51) and 3,291 ng/mL (0.40), respectively. The treatment was discontinued immediately after the diagnosis of medulloblastoma. Approximately 1.2 years later, death of the patient was confirmed during the off-treatment follow-up process.

### Adverse events of interest

The incidence of AEs of interest was analyzed for the whole study period. Fourteen neoplasms (191 per 100,000 PY) were diagnosed in 11 patients (6 females, 5 males): 5 benign (68 per 100,000 PY) and 9 malignant (123 per 100,000 PY) (Table 3). Seven cases out of total 9 malignant neoplasms were recurrence cases in craniopharyngioma, diagnosed solely in OGHD patients, 2–6 years after the diagnosis of primary tumors and 8 months to 5 years after rhGH treatment initiation. Four neoplasms, three malignant and one benign, were considered as ADRs (Table 4). Ovarian dysgerminoma was reported in 9 years old TS (45,X/46,XY) patient during the rhGH treatment (approximately one year after the start of the rhGH treatment). The treatment was discontinued immediately after the diagnosis and she recovered after receiving chemotherapy.

**Table 3:** Incidence of adverse events of interest during whole study period.

Variable	Total (n = 2,024)^a^	IGHD (n = 1,189)	OGHD (n = 107)	TS (n = 197)	SGA (n = 206)	ISS (n = 315)	CRF (n = 9)
Death	1 (14)	1 (23)	-	-	-	-	-
All neoplasms	14 (191)	4 (93)	7 (1289)	3 (255)	-	-	-
Malignancy	9 (123)	1 (23)	7 (1289)	1 (85)	-	-	-
Benign	5 (68)	3 (70)		2 (170)	-	-	-
Hypothyroidism	22 (300)	6 (140)	3 (552)	11 (937)	-	1 (118)	1^†^
Glucose intolerance^*^	7 (95)	2 (47)	3 (552)	-	1 (218)	1 (118)	-
Scoliosis	11 (150)	7 (163)	1 (184)	2 (170)	1 (218)	-	-
Benign intracranial hypertension	1 (14)	1 (23)	-	-	-	-	-
Pancreatitis	1 (14)	1 (23)	-	-	-	-	-
Fluid retention	1 (14)	1 (23)	-	-	-	-	-
Gynecomastia	1 (14)	-	-	-	-	1 (118)	-
Sleep apnea syndrome	1 (14)	1 (23)	-	-	-	-	-

a Addition of each subgroup number does not sum up to 2,024 as one patient with GHD could not be classified as either IGHD or OGHD. The patient with non-specified GHD etiology did not experience any adverse event. Data show absolute numbers (rates per 100,000 patient-years).

^*^Includes diabetes mellitus (n = 3), hyperglycemia (n = 3) and glucose tolerance impaired (n = 1),

^†^Due to small sample size, rates per 100,000 patient-years of chronic renal failure cohort was not analyzed separately.

*CRF* chronic renal failure, *GHD* growth hormone deficiency, *IGHD* idiopathic growth hormone deficiency, *ISS* idiopathic short stature, *OGHD* organic growth hormone deficiency, *SGA* small for gestational age, *TS* Turner syndrome.

**Table 4:** Neoplasms reported during whole study period.

Indication for rhGH treatment	Sex	Age at the baseline (years)	Age at neoplasm diagnosis (years)	Neoplasm type	rhGH at the time of diagnosis	Relationship with rhGH	Action undertaken	Outcome
Malignant								
Organic GHD	female	19	21	craniopharyngioma recurrence^*^^,^^†^	yes	possible	rhGH stopped	ongoing
Organic GHD	male	9	14	craniopharyngioma recurrence^†^	yes	unlikely	rhGH interrupted	resolved
			17	craniopharyngioma recurrence^†^^,^^‡^	yes	unlikely	none	resolved
Organic GHD	female	13	14	craniopharyngioma recurrence^*^^,^^†^	yes	possible	rhGH stopped	resolved
			15	craniopharyngioma recurrence^†^	yes	unlikely	none	resolved
			17	craniopharyngioma recurrence^†^^,^^§^	off-treatment	unlikely	none	resolved
Organic GHD	male	7	8	craniopharyngioma recurrence^*^^,^^†^	off-treatment	possible	rhGH stopped	resolved
Idiopathic GHD	male	12	13	medulloblastoma^†^	yes	unlikely	rhGH stopped	death
TS	female	8	9	ovarian dysgerminoma stage unspecified^†^	yes	not related	rhGH interrupted	resolved
Benign								
Idiopathic GHD	female	9	9	skin papilloma^*^	yes	possible	none	resolved
Idiopathic GHD	male	6	8	skin papilloma	yes	unlikely	none	resolved
Idiopathic GHD	male	3	11	skin papilloma	yes	not related	none	resolved
TS	female	4	11	neurofibroma	yes	not related	none	ongoing
TS	female	10	13	osteochondroma	yes	not related	none	ongoing

^*^Evaluated as related to rhGH treatment,

^†^reported as SAE,

^‡^Epilepsy was reported as AE approximately 2 years after this was resolved (ongoing).

^§^CSF leak was reported as AE 6 days after this was resolved (resolved after 9 days).

*GHD* growth hormone deficiency, *rhGH* recombinant human growth hormone, *TS* Turner syndrome

Hypothyroidism (22 cases, 300 per 100,000 PY) was reported primarily in TS (11 cases) and GHD cohorts (9 cases; 6 patients with IGHD and 3 with OGHD), typically during treatment (21 cases) and mostly in female patients (19 cases). Median time elapsed from GH treatment onset -to hypothyroidism diagnosis was 1.9 (IQR: 0.8, 2.7) years. More than 50% of the cases were considered to be unrelated to rhGH, and 10 (136 per 100,000 PY) were considered as ADRs.

There were seven cases (95 per 100,000 PY) of glucose intolerance (Table 3). Of these, four cases were regarded as ADRs, including type 2 diabetes mellitus (T2DM, 2 cases), glucose tolerance impaired (1 case), and hyperglycemia (1 case). In case of new-onset T2DM, three cases were detected during the whole follow-up period (Table 5). In 3 out of 7 glucose intolerance cases, hyperglycemia (2 cases) and glucose tolerance impaired (1 case), have been resolved.

**Table 5:** New-onset diabetes cases reported during whole study period.

Type of DM	Indication for rhGH treatment	Age at the baseline (years)	Age at DM diagnosis (years)	Additional risk factors	BMI (SDS) at the baseline	rhGH at the time of diagnosis	Relationship with rhGH	Action undertaken	Outcome
Type 2	Idiopathic GHD	9	11	Family history (+)	1.98	yes	possible	none	ongoing
Type 2	Organic GHD	16	17	-	2.27	yes	not related	none	ongoing
Type 2	Organic GHD	11	15	-	1.47	yes	probable	rhGH interrupted	ongoing

*DM* Diabetes mellitus, *BMI* body mass index, *GHD* growth hormone deficiency, *rhGH* recombinant human growth hormone

Scoliosis (11 cases, 150 per 100,000 PY) was reported in SGA (1 case), TS (2 cases) and GHD cohorts (8 cases; 7 patients with IGHD and 1 with OGHD) during treatment. Median time elapsed from GH treatment onset -to scoliosis diagnosis was 2.3 (IQR: 1.1, 3.2) years. Five cases (68 per 100,000 PY) were considered as ADRs.

### Laboratory results

**Fig 2:**
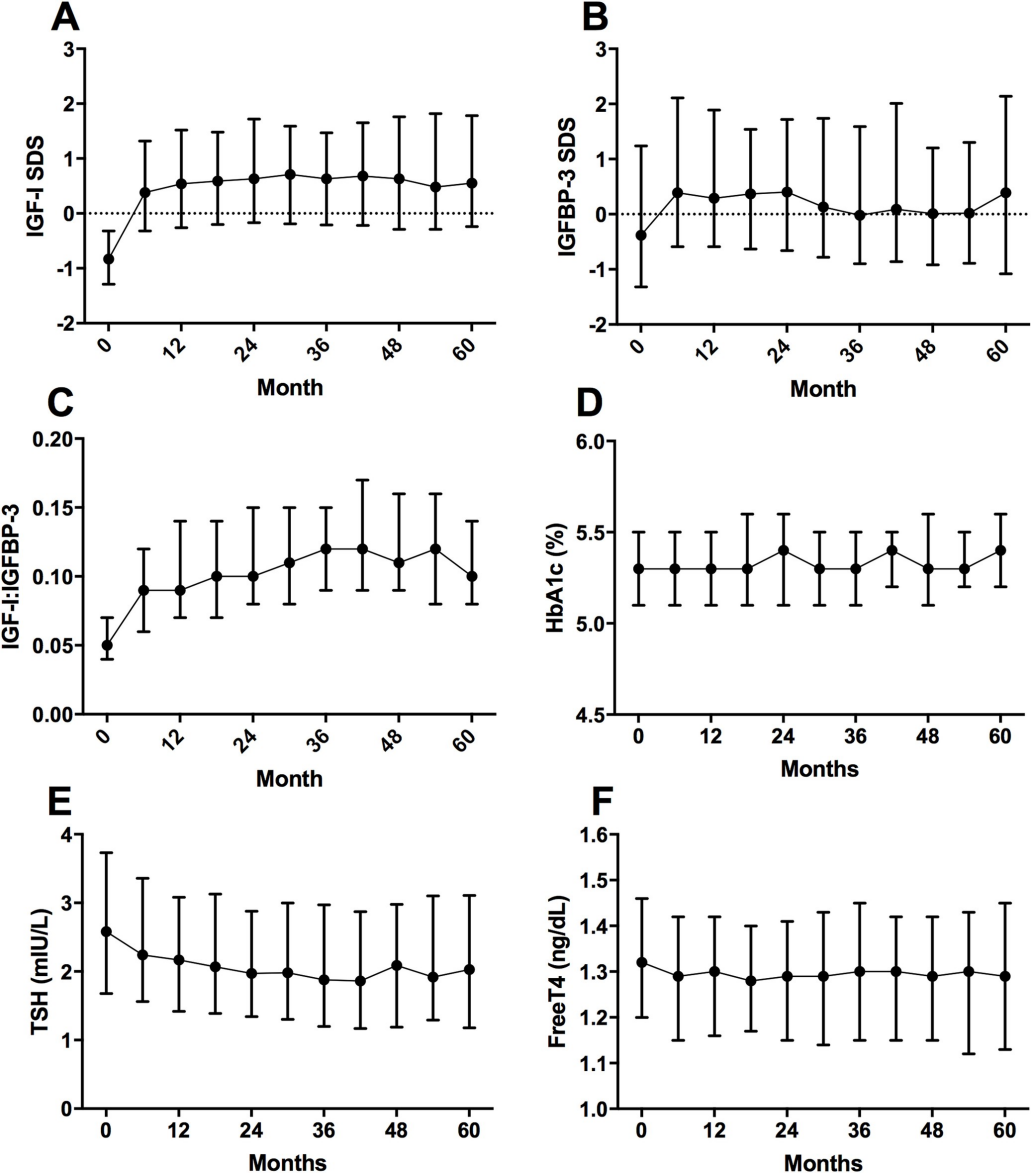
Laboratory tests for IGF-I, IGFBP-3, HbA1c, TSH, and free T4 (safety set). The symbols represent median values and the vertical bars indicate interquartile range.

**Fig 3:**
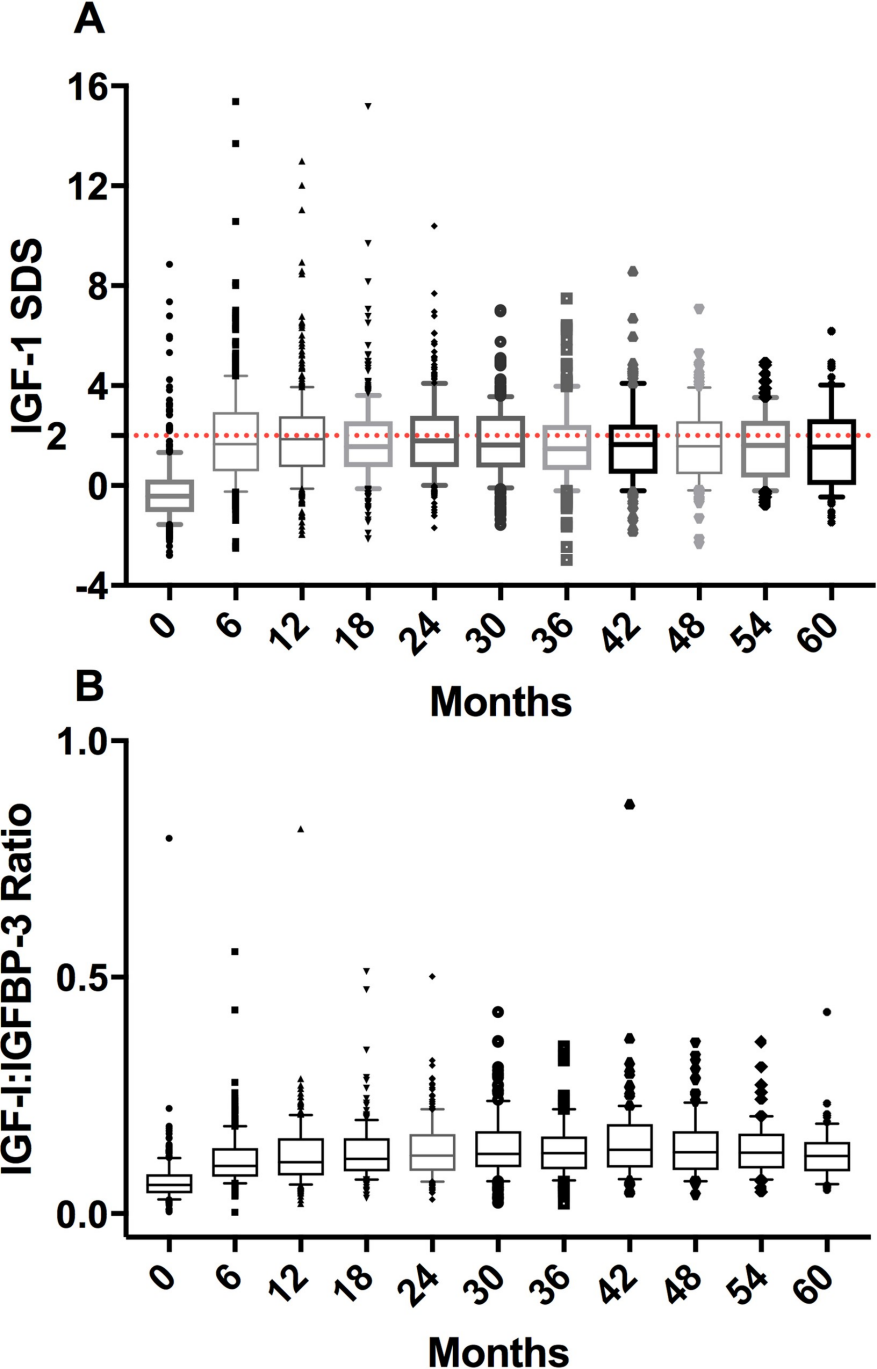
IGF-I SDS. (A) and IGF-I to IGFBP-3 ratio (B) in a subgroup of patients with supra-physiological level of IGF-I. The boxes represent interquartile ranges and the whiskers represent 10^th^ and 90^th^ percentiles. (A) In the subgroup of patients having supra-physiological level of IGF-I, some were observed to have extremely high SD scores of IGF-I since the initiation of rhGH treatment; however, such extreme values tended to disappear as treatment progressed. (B) IGF-I to IGFBP-3 ratio stayed relatively stable since 6 months of treatment.

Irrespective of the cohort, IGF-I SDS and IGFBP-3 SDS significantly increased at six months after rhGH treatment (*p*<0.001), and their levels remained at a plateau thereafter (Fig 2). Supraphysiological levels of IGF-I (IGF-I SDS >+2) were found at least once in 508 patients (25.1%), more frequently within one year of treatment and in TS cohort (82/197 patients; Fig 3). Median IGF-I to IGFBP-3 ratio remained at a stable level of 0.1. The subgroup with at least one IGF-I SDS value >+2 did not differ from the other patients in terms of AE (15,027 vs. 29,197 per 100,000 PY) and SAE frequencies (1,940 vs. 2,527 per 100,000 PY), but more often presented with serious ADRs (226 vs. 181 per 100,000 PY).

Other laboratory findings included a slight decrease in TSH levels, observed until 42 months of treatment. No significant changes were found in fT4 and HbA1c levels (Fig 2).

### Effectiveness results

**Fig 4:**
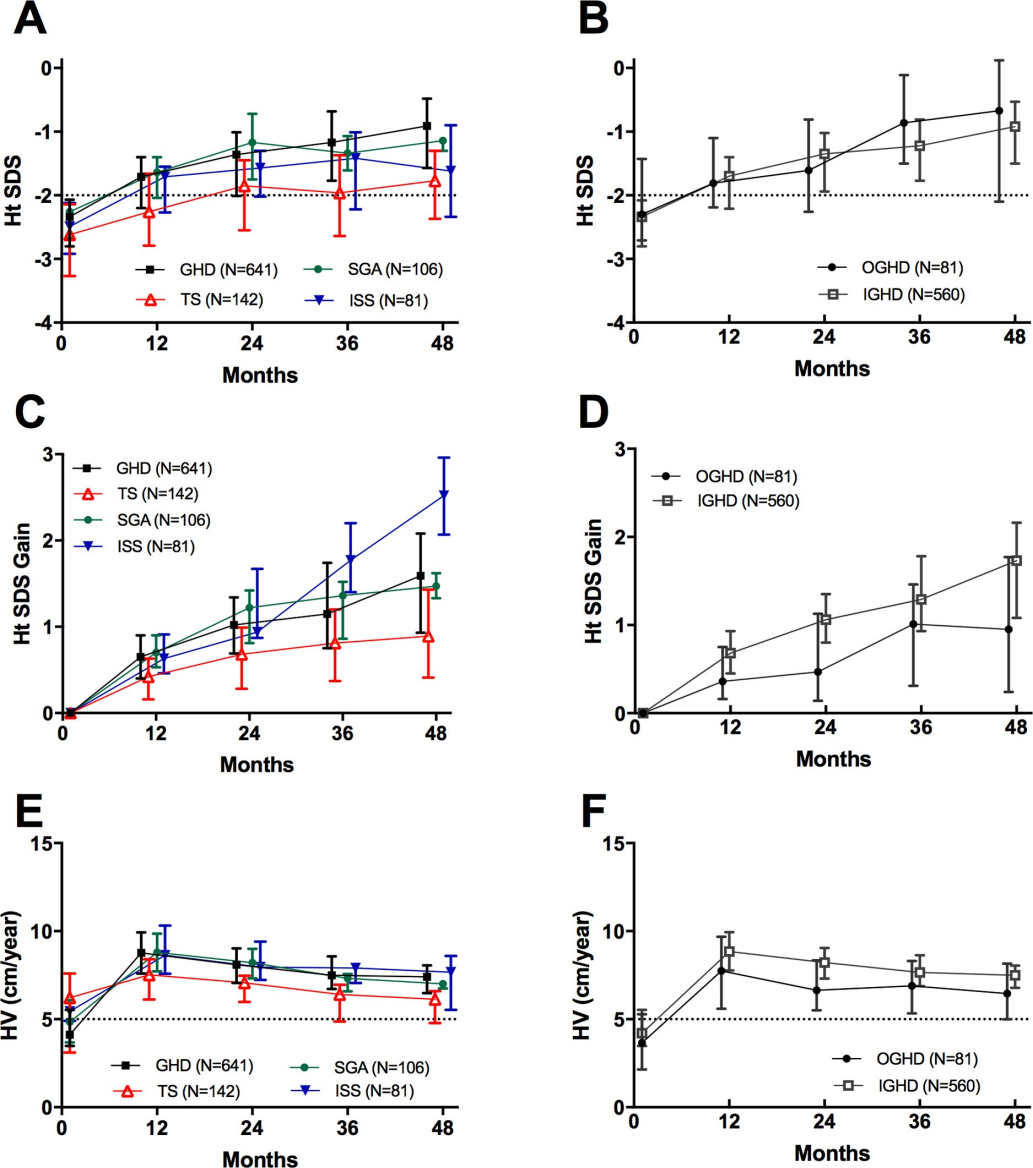
Auxological measurements through the fourth year. The symbols indicate the median values and the vertical bars indicate interquartile range. IGHD was confirmed by at least two GH stimulation tests (peak responses from both tests <10 ng/mL).

Median (IQR) height SDS for patients included in the effectiveness set was -2.37 (-2.89, -2.07, n = 976) at the baseline, and -1.39 (-2.05, -0.87, n = 243) and -1.30 (-1.99, -0.62, n = 148) at 36 and 48 months of treatment, respectively. Ht SDS at six months of treatment was significantly higher (*p*<0.001) than at the baseline in GHD, ISS, TS and SGA cohorts. In GHD cohort, an increase in height SDS continued until the fourth year, and in the other cohorts, until the second year (Fig 4). After 4 years of GH treatment, mean changes (95% CI) in Ht SDS in GHD (n = 95), TS (n = 44) and SGA cohorts (n = 5) were 1.52 (1.32, 1.73), 0.86 (0.64, 1.08) and 1.27 (0.53, 2.00), respectively. In GHD subgroups, mean Ht SDS gain (95% CI) at 4 years was 1.68 (1.49, 1.87) in IGHD (n = 71) and 1.06 (0.48, 1.64) in OGHD (n = 24).

At six months of treatment, mean (95% CI) HV was 9.20 (9.02, 9.38), being nearly twice as high as at the baseline. Most patients achieved the highest HV during the first year of treatment (Fig 4). At 12 months, the highest HV was documented in ISS cohort (mean: 8.81, 95% CI: 8.31, 9.32) and the lowest in TS cohort (mean: 7.27; 95% CI: 6.94, 7.60). In GHD, SGA and ISS cohorts, mean HV was maintained above 7 cm per year until the second year and then decreased gradually, down to 5.30–5.74 cm annually at four years. Irrespective of the study timepoint, HV for TS cohort was lower than for other cohorts (Fig 4).

## Discussion

According to a general consensus, rhGH can be safely administered for patients without known risk factors of malignant neoplasia, but the available evidence in this matter is still inconclusive. Recently, SAGhE study demonstrated a higher incidence of bone and bladder neoplasms in the rhGH-treated cohort, but without enough evidence for a causal role of the agent [19]. In our study, 9 malignant neoplasms were diagnosed in 6 patients (crude incidence: 0.3%, 123 per 100,000 PY). After excluding seven cases of craniopharyngioma recurrence, primary malignant neoplasia incidence was 0.1% (27 per 100,000 PY); in the investigators’ causality assessment, none were rhGH-related. The incidence of malignant neoplasms was slightly higher than in the previous studies, KIGS and NCGS (16.4 and 5.8 per 100,000 PY, respectively) [20,21]. However, the latter two studies were markedly larger than LGS (58,603 and 33,161 patients participated in KIGS and NCGS, respectively). Furthermore, it should be emphasized that the neoplasia risk in rhGH-treated patients is known to decrease with follow-up time, and both KIGS and NCGS were based on more than 20-year accumulated data, as compared with only a 5-year dataset in our study. Hence, conclusions about cancer risk in the South Korean pediatric population treated with rhGH should not be formulated earlier than at the end of the designated follow-up period for all targeted patients.

One patient died during off-treatment follow-up period after being diagnosed with medulloblastoma (mortality: 0.05%, 14 per 100,000 PY). A contribution of rhGH therapy was considered unlikely in this case. Medulloblastoma is one of the most common pediatric cancers [22], and previous studies found no correlation between rhGH treatment and progression or recurrence of brain tumors [6,23,24].

The incidence of AEs associated with glucose intolerance in LGS patients was 0.3% (95 per 100,000 PY), and T2DM was diagnosed in 0.15% of the study participants. While the risk of glucose intolerance and T2DM in rhGH-treated patients with GHD and ISS is low, those who were treated with rhGH for TS or SGA are more prone to metabolic diseases than individuals from the general population [6,25]. However, it is unclear whether the increased metabolic risk in this group is directly related to the treatment, or is rather a consequence of underlying conditions. In a previous study, 199 SGA patients treated with rhGH were followed-up for 5 years; both insulin resistance and beta cell dysfunction caused by rhGH have been resolved within 6 months post-treatment, and at the end of the follow-up period, body composition, insulin sensitivity and beta-cell function did not differ between the study group and the untreated controls [26]. This implies that metabolic AEs related to rhGH treatment are reversible and probably clinically irrelevant.

Our cohort included 22 patients who developed hypothyroidism during rhGH treatment (incidence: 1.1%, 300 per 100,000 PY). Most patients who developed hypothyroidism received rhGH due to TS (n = 11) or GHD (n = 9). Hypothyroidism is a common comorbidity in TS patients, occurring regardless of rhGH treatment [27]; indeed, in 8 out of 11 patients with TS included in LGS study, the investigators evaluated hypothyroidism as not related to rhGH therapy.

Scoliosis has been observed in 11 patients (incidence: 0.5%, 150 per 100,000 PY). Among them, two were patients with TS, in which condition scoliosis is known to be more prevalent even without rhGH treatment [6,28]. As the rapid growth during rhGH treatment may accelerate the progression of scoliosis, regular radiographic check-up is recommended for those receiving rhGH therapy [1,6].

One of the concerns related to administration of rhGH at higher doses is potential risk associated with the supraphysiological level of IGF-I. In our study, 25% of patients at least once had IGF-I SDS >+2, which corresponds to the supraphysiological level of this compound. However, in most of these patients, the elevated levels of IGF-I were observed temporarily, usually at early stages of the treatment, and then remained within the recommended range. Although serious ADR rate in patients with IGF-I SDS >+2 was slightly higher than in those with lower IGF-I SDS values, none of them was withdrawn from the study prematurely. The increase in IGF-I SDS was associated with a concomitant increase in IGFBP-3 SDS and therefore, IGF-I to IGFBP-3 ratio remained at a relatively stable level. Thus, it may be presumed that the increase in IGF-I level was counterbalanced by IGFBP-3. The increase in IGF-I level is generally known to be proportional to rhGH dose. The TS cohort, in which median dose of rhGH was the highest, contained also the largest proportion of patients with IGF-I SDS >+2. In International Outcome Study, including 13,843 patients receiving Norditropin, no significant correlation was found between the rhGH dose and AE rate [29], which is consistent with our findings.

While the treatment outcomes were most favorable in patients with complete GHD, individuals with TS seemed not to benefit fully from rhGH therapy. In up to 61% of patients for TS cohort, Ht SDS at one-year follow-up was no greater than 0.5. A previous study demonstrated that the outcomes of rhGH treatment are predicted by its dose, age at the therapy onset, mid-parental height and growth response within the first year [30]. The 5-year overall median dose of rhGH in our TS cohort was 0.31 mg/kg/week; this shows more than a half of the patients received rhGH at a slightly lower dose than recommended (0.33 mg/kg/week). Another potential reason for less satisfactory growth outcomes in TS patients might be their relatively older age at treatment initiation (median 9.49 years). Although no strict guideline exists in this matter, it is generally accepted that the earlier the rhGH treatment implemented, the better its outcome.

More than 50% of patients considered in the safety set were excluded from the effectiveness analysis. The largest proportion of the excluded patients did not satisfy the indication requirements, especially for IGHD (25.8%) and ISS (55.9%). This implies that regardless of the reimbursement policies and commonly accepted clinical guidelines, there might be a parental pressure to prescribe rhGH products; indeed, such a tendency was documented in one study conducted in the South Korean population [31]. The rationale for the implementation of rhGH treatment in all ISS patients raises controversies and hence, the decision to start the therapy in this group should be based on comprehensive evaluation of several factors, among them quality of life-related issues, economic burden and functional impact, rather than solely on height benefit and parental request [32].

The results presented above may have a few limitations inherent to each registry study. This study is an observational study without an untreated control group. Moreover, laboratory parameters, such as IGF-I and IGFBP-3, were measured at each site, which might contribute to an interlaboratory measurement bias. To overcome these limitations and to minimize the risk of bias due to missing data, we used a systematic approach to data collection. Many registry studies are based on voluntary reporting of AEs, and/or only ADRs are registered. Meanwhile, our patients were seen at participating centers every six months, to collect all AE data and physical measurements in a standardized manner.

## Conclusions

The spectrum of AEs present in rhGH-treated Korean patients was similar to previously reported for other populations. The incidence of AEs, including adverse events of interest, such as neoplasia, glucose intolerance and hypothyroidism, was low. No alarming safety signal was identified at the time of the interim analysis. Most of the growth-retarded patients showed continuous improvement in their Ht SDS during the follow-up period, with the most prominent effect observed within a year of treatment initiation. The beneficial effect of rhGH on Ht SDS gain was maintained for up to four years.

## Supporting information

S1 Table List of institutional review boards that approved the study. (DOCX)

S2 Table Baseline demographic characteristics of patients included in the effectiveness set. (DOCX)
