# Supplementary material for: Long-term safety and effectiveness of growth hormone therapy in Korean children with growth disorders: 5-year results of LG Growth Study
Source: PLoS One. 2019 May 16;14(5):e0216927. doi: 10.1371/journal.pone.0216927 (PMC6522217; doi:10.1371/journal.pone.0216927)
Supplement: S1 Table — (DOCX) [file pone.0216927.s001.docx]

S1 Table. List of Institutional Review Boards that approved the study protocol

| No. | IRB | Note |
| --- | --- | --- |
| 1 | Ajou University Hospital Institutional Review Board |  |
| 2 | Asan Medical Center Institutional Review Board |  |
| 3 | Boramae Hospital Institutional Review Board |  |
| 4 | Bundang Jeseang General Hospital Institutional Review Board |  |
| 5 | Catholic Kwandong University International St. Mary’s Hospital Institutional Review Board |  |
| 6 | CHA Gangnam Medical Center, CHA University, Institutional Review Board |  |
| 7 | CHA University CHA Bundang Medical Center Institutional Review Board |  |
| 8 | Cheil General Hospital Institutional Review Board |  |
| 9 | Chonbuk National University Hospital Institutional Review Board |  |
| 10 | Chonnam National University Hospital Institutional Review Board |  |
| 11 | Chosun University Hospital Institutional Review Board |  |
| 12 | Chungbuk National University Hospital Institutional Review Board |  |
| 13 | Chungnam National University Hospital Institutional Review Board |  |
| 14 | Daegu Catholic University Medical Center Institutional Review Board |  |
| 15 | Daegu fatima Hospital Institutional Review Board |  |
| 16 | Dankook University Hospital Institutional Review Board |  |
| 17 | Dong-a University hospital Institutional Review Board |  |
| 18 | Dongguk University Ilsan Hospital Institutional Review Board |  |
| 19 | Eulji General Hospital Institutional Review Board |  |
| 20 | Eulji University Hospital Institutional Review Board |  |
| 21 | Ewha Womans University Medical Center Institutional Review Board |  |
| 22 | Hallym University Chuncheon Sacred Heart Hospital Institutional Review Board |  |
| 23 | Hallym University Chuncheon Sacred Heart Hospital Institutional Review Board* | Resubmitted |
| 24 | Hallym University Dongtan Sacred Heart Hospital Institutional Review Board |  |
| 25 | Hallym University Kangnam Sacred Heart Hospital Institutional Review Board |  |
| 26 | Hallym University Scared Heart Hospital Institutional Review Board |  |
| 27 | Hanyang University Guri Hospital Institutional Review Board |  |
| 28 | Hanyang University Guri Hospital Institutional Review Board* | Resubmitted |
| 29 | Inha University Hospital Institutional Review Board |  |
| 30 | Inje University Busan Paik Hospital Institutional Review Board |  |
| 31 | Inje University Haeundae Paik Hospital Institutional Review Board |  |
| 32 | Inje University Ilsan Paik Hospital Institutional Review Board |  |
| 33 | Inje University Sanggye Paik Hospital Institutional Review Board |  |
| 34 | Jeju National University Hospital Institutional Review Board |  |
| 35 | Kangdong Sacred Heart Hospital Institutional Review Board |  |
| 36 | Kangwon National University Hospital Institutional Review Board |  |
| 37 | Kangwon National University Hospital Institutional Review Board* | Resubmitted |
| 38 | Keimyung University Dongsan Hospital Institutional Review Board |  |
| 39 | Konkuk University Medical Center Institutional Review Board |  |
| 40 | Konyang University Hospital Institutional Review Board |  |
| 41 | Korea Institute of Radiological & Medical Sciences Institutional Review Board |  |
| 42 | Korea University Anam Hospital Institutional Review Board |  |
| 43 | Korea University Ansan Hospital Institutional Review Board |  |
| 44 | Korea University Guro Hospital Institutional Review Board |  |
| 45 | Kosin University Gospel Hospital Institutional Review Board |  |
| 46 | Kwangju Christian Hospital Institutional Review Board |  |
| 47 | Kyung Hee University Hospital at Gangdong Institutional Review Board |  |
| 48 | Kyungpook National University Chilgok Hospital Institutional Review Board |  |
| 49 | Kyungpook National University Hospital Institutional Review Board |  |
| 50 | Myongji Hospital Institutional Review Board |  |
| 51 | National Medical Center Institutional Review Board |  |
| 52 | Pusan National University Hospital Institutional Review Board |  |
| 53 | Pusan National University Yangsan Hospital Institutional Review Board |  |
| 54 | Samsung Medical Center Institutional Review Board |  |
| 55 | Seoul National University Hospital Institutional Review Board |  |
| 56 | Soon Chun Hyang University Hospital Bucheon Institutional Review Board |  |
| 57 | Soon Chun Hyang University Hospital Seoul Institutional Review Board |  |
| 58 | The Catholic University of Korea, Bucheon ST. Mary’s Hospital Institutional Review Board |  |
| 59 | The Catholic University of Korea, Seoul ST. Mary’s Hospital Institutional Review Board |  |
| 60 | The Catholic University of Korea, ST. Vincent’s Hospital Institutional Review Board |  |
| 61 | The Catholic University of Korea, Yeouido ST. Mary’s Hospital Institutional Review Board |  |
| 62 | Ulsan University Hospital Institutional Review Board |  |
| 63 | Wonju Severance Christian Hospital Institutional Review Board |  |
| 64 | Wonkwang University Hospital Institutional Review Board |  |
| 65 | Wonkwang University Hospital Institutional Review Board* | Resubmitted |
| 66 | Yonsei University Gangnam Severance Hospital Institutional Review Board |  |
| 67 | Yonsei University Severance Hospital Institutional Review Board |  |
| 68 | Yonsei University Wonju Severance Christian hospital Institutional Review Board |  |
| 69 | NA** | Non-IRB |
| 70 | NA** | Non-IRB |
| 71 | NA** | Non-IRB |
| 72 | NA** | Non-IRB |
| 73 | NA** | Non-IRB |

*In four sites, IRB reviews were repeated for the same protocol because of an administrational procedure (due to change of the principal investigator).

**Five sites did not have IRB.
