# Supplementary material for: Long-term safety and effectiveness of growth hormone therapy in Korean children with growth disorders: 5-year results of LG Growth Study
Source: PLoS One. 2019 May 16;14(5):e0216927. doi: 10.1371/journal.pone.0216927 (PMC6522217; doi:10.1371/journal.pone.0216927)
Supplement: S2 Table — (DOCX) [file pone.0216927.s002.docx]

**S2 Table.** **Baseline demographic characteristics of patients included in the effectiveness set**

| Variable | Total  (n=978) | GHD  (n=641) | TS  (n=142) | SGA  (n=106) | ISS  (n=81) | CRF  (n=8) |
| --- | --- | --- | --- | --- | --- | --- |
| Sex: |  |  |  |  |  |  |
| male | 486  (49.69%) | 387  (60.37%) | 0  (0.00%) | 49  (46.23%) | 45  (55.56%) | 5  (62.50%) |
| female | 492  (50.31%) | 254  (39.63%) | 142  (100.0%) | 57  (53.77%) | 36  (44.44%) | 3  (37.50%) |
| Age (years) | 8.49  (2.04, 19.23) | 8.52  (2.12, 19.23) | 9.49  (2.04, 18.58) | 6.94  (3.36, 14.18) | 8.10  (2.38, 15.60) | 9.59  (2.48, 15.88) |
| Puberty^a^ |  |  |  |  |  |  |
| pre-pubertal | 398  (83.79%) | 239  (85.36%) | 76  (80.00%) | 49  (87.50%) | 32  (76.19%) | 2  (100.0%) |
| pubertal | 77  (16.21%) | 41  (14.64%) | 19  (20.00%) | 7  (12.50%) | 10  (23.81%) | 0  (0.00%) |
| BA (years) | 6.5  (0.6, 17.0) | 6.0  (0.6, 17.0) | 8.5  (1.0, 13.5) | 6.0  (1.5, 13.3) | 7.0  (2.3, 13.5) | 8.85  (2.0, 13.5) |
| BA-CA (years) | -1.59  (-6.04, 4.49) | -1.84  (-6.04, 2.71) | -0.93  (-5.75, 2.53) | -1.06  (-4.67, 2.27) | -1.36  (-4.60, 4.49) | -2.44  (-4.87, 0.30) |
| Height SDS | -2.37  (-6.97, 2.04) | -2.34  (-6.97, 2.04) | -2.58  (-6.34, -0.78) | -2.25  (-5.14, -1.89) | -2.51  (-5.31, -1.82) | -2.43  (-3.54, 0.00) |
| Target height SDS (MPH) | -0.69  (-3.58 ,1.48) | -0.75  (-3.58 ,1.48) | -0.15  (-2.78 ,1.20) | -0.85  (-3.35 ,0.90) | -0.96  (-2.34 ,0.47) | -0.33  (-1.23 ,0.20) |
| BMI SDS | -0.21  (-5.60, 3.4) | -0.15  (-3.90, 2.8) | 0.37  (-1.92, 3.47) | -0.93  (-5.60, 2.09) | -0.59  (-4.60, 1.69) | -0.89  (-2.89, 1.61) |
| rhGH dose^b^  (mg/kg/week) |  |  |  |  |  |  |
| Daily | 0.25  (0.03, 0.59) | 0.23  (0.04, 0.5) | 0.31  (0.18, 0.43) | 0.28  (0.03, 0.59) | 0.26  (0.17, 0.45) | 0.29  (0.23, 0.33) |
| Weekly | 0.60  (0.32, 2.50) | 0.60  (0.32, 2.50) | - | - | - | - |

a Some patients’ puberty data are missing and only available data are accounted. b 4-year overall median dose.

Data show numbers (%) or medians (min, max). In GHD and ISS cohorts, only patients with the results of growth hormone stimulation test were included in the effectiveness set.

*BA* bone age, *BMI* body mass index, *CA* chronological age, *CRF* chronic renal failure, *GHD* growth hormone deficiency, *ISS* idiopathic short stature, *MPH* mid parental height, *rhGH* recombinant human growth hormone, *SDS* standard deviation score, *SGA* small for gestational age, *TS* Turner syndrome
